# Evaluation of Hippocampal Microanatomy and Neuro-Biomarkers Following Administration of Silver Nanoparticles Conjugated with Tenofovir Disoproxil Fumarate in Experimental Diabetic Rats

**DOI:** 10.3390/ph17121635

**Published:** 2024-12-05

**Authors:** Sodiq Kolawole Lawal, Samuel Oluwaseun Olojede, Babatunde Adebola Alabi, Kafalotse Sylvia Dithole, Samuel Thopho Matula, Edwin Coleridge Naidu, Carmen Olivia Rennie, Onyemaechi Okpara Azu

**Affiliations:** 1 School of Nursing, Faculty of Health Sciences, University of Botswana, Private Bag UB 0022, Plot 4775, Notwane Road, Gaborone, Botswana; ditholek@ub.ac.bw (K.S.D.); matulast@ub.ac.bw (S.T.M.); 2 Discipline of Clinical Anatomy, School of Laboratory Medicine & Medical Sciences, Nelson R Mandela School of Medicine, University of KwaZulu-Natal, Durban 3629, South Africa; naidue@ukzn.ac.za (E.C.N.); rennie@ukzn.ac.za (C.O.R.); 3 Division of Human Anatomy, Department of Human Biology, Faculty of Medicine and Health Sciences, Walter Sisulu University, Nelson Mandela Drive, Mthatha 5117, South Africa; solojede@wsu.ac.za; 4 Department of Pharmacology and Therapeutics, Bowen University, Iwo P.O. Box 284, Nigeria; babatunde.alabi@bowen.edu.ng; 5 Department of Medical Biosciences, University of the Western Cape, Bellville 7535, South Africa; oazu@uwc.ac.za

**Keywords:** tenofovir, silver nanoparticles, neurotoxicity, metabolic disorder, microanatomy, diabetes

## Abstract

Adverse complications like metabolic disorders, neurotoxicity, and low central nervous system (CNS) penetration are associated with the long-term use of tenofovir disoproxil fumarate (TDF). Therefore, some modifications are required to enhance neurological functions using silver nanoparticles (AgNPs). This study aimed to evaluate the neuroprotective impact of silver nanoparticles (AgNPs)-conjugated TDF as AgNPs-TDF on the hippocampal microanatomy and some neuro-biomarkers of diabetic rats. Forty-two male Sprague-Dawley rats, with an average weight of 250 ± 13 g, were divided into non-diabetic and diabetic groups. They were further divided into 3 groups each (n = 7): non-diabetic control (NC), non-diabetic + TDF (NTF), and non-diabetic + TDF + silver nanoparticles (NTS), as well as diabetic control (DC), diabetic + TDF (DTF), and diabetic + TDF + silver nanoparticles (DTS). The characterization of AgNPs-TDF was assessed, and the conjugates were administered to the diabetic rats, followed by behavioral testing and biochemical, immunohistochemical, and microanatomy analyses of the hippocampus. The results showed that the administration of AgNPs-TDF significantly reduced the blood glucose level, malondialdehyde (MDA), and inflammatory biomarker concentrations in DTS compared with the DTF and DC groups. Furthermore, AgNPs-TDF administration significantly increased the levels of tissue superoxide dismutase (SOD), reduced glutathione (GSH), and insulin-like growth factor-1 in DTS compared with the DTF and DC groups. In addition, the DTS group revealed a monomorphic pattern of dark-stained neuronal nuclei similar to the control group and showed neuroprotective effects on hippocampal microanatomy compared with the DTF group. This study shows that AgNPs-TDF restores various alterations in the hippocampus and improves cognitive functions in diabetic rats.

## 1. Introduction

The inception of combined antiretroviral therapy (cART) has benefited people living with HIV, and such benefits achieved with prolonged administration of cART include reduced mortality, morbidity, and opportunistic infection, immune system enhancement, viral load suppression, and cluster of differentiation 4 cell count (CD4 count) improvement [1,2]. Despite these better outcomes, long-term administration of cART has been linked with severe metabolic disorders and neurotoxicity [3,4]. Especially, some antiretroviral drugs belonging to nucleoside reverse transcriptase inhibitors (NRTIs), such as abacavir, stavudine, emtricitabine, lamivudine, and tenofovir disoproxil fumarate (TDF), have been linked with neurotoxicity [5].

Tenofovir disoproxil fumarate is a widely used drug either alone or in combination with other antiretroviral drugs as a cocktail known as highly active antiretroviral therapy (HAART) for managing HIV and AIDS [6,7]. TDF alone is often used as pre-exposure prophylaxis (PrEP) by HIV-negative individuals and sex workers because it is highly effective at reducing the risk of sexual acquisition or transmission of HIV [7,8]. A study has reported that TDF’s oral administration in combination with emtricitabine (FTC) is the most effective PrEP for HIV prevention compared with other combinations [9]. Furthermore, statistics have shown that about 1.5 million people were placed on TDF-based oral prophylaxis therapy in 2021, leading to the global decline in the spread of HIV [8]. Despite the advantages of TDF, studies have associated TDF with severe metabolic disturbances, neurotoxicity, and mitochondrial dysfunction [3,4,6]. Several studies have reported the association of long-term exposure to TDF with glucose metabolism disorders, such as insulin resistance and diabetes mellitus [10,11]. The mechanism of TDF-induced neurotoxicity has been linked to mitochondrial toxicity and inhibition of DNA polymerase γ resulting from increased oxidase stress [12,13].

In addition to the neurotoxicity associated with TDF, empirical evidence has described TDF as having low CNS penetration amongst the NRTIs [14]. In addition, a similar study has reported only a low concentration of tenofovir in the cerebrospinal fluid (CSF), among other antiretroviral drugs [15]. Thus, the quantity of TDF in the CNS may not be sufficient to reduce the viral loads but enough to cause neurotoxicity [14,16].

Recently, attention has been drawn to the use of nanoparticle drug delivery vehicles to navigate biological barriers and deliver antiretroviral drugs to anatomical sanctuary sites, such as the CNS, testicular tissue, and lymphoid organs, which harbor HIV [17]. Among several nanoparticles that have been widely employed, silver nanoparticles are currently receiving attention as potential agents in delivering cART to nervous tissues owing to their tunable shape ability [16,18,19]. In addition, silver nanoparticles are utilized in biomedical research to deliver therapeutic agents across the CNS due to their unique properties, such as anti-inflammatory, antidiabetic, antiviral, and antioxidant, which are vital to the alleviation of HIV-associated neurocognitive disorders and toxicity associated with antiretroviral drugs [19,20,21,22]. Based on these reports, we speculated that silver nanoparticle–TDF conjugates could protect the hippocampus against TDF-associated neurotoxicity via inhibition of inflammatory mediator release, oxidative stress, hippocampal neuronal cell membrane integrity alteration, hippocampal nerve organelle disintegration, hippocampal astrocytes, and glial cell degeneration.

**Figure 1:**
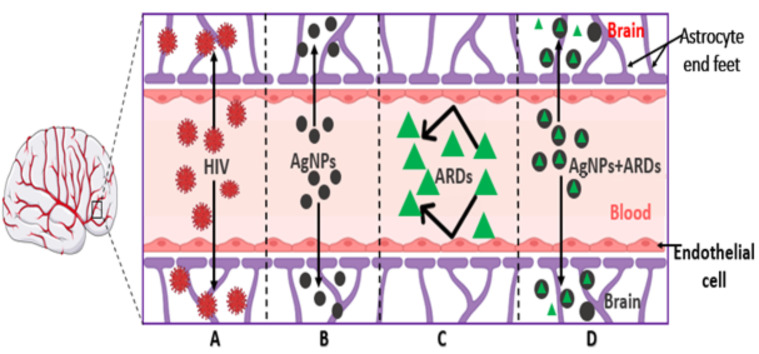
Illustration of the permeability of the blood–brain barrier to (**A**) HIV, (**B**) silver nanoparticles, (**C**) ARDs/TDF, and (**D**) AgNPs + ARDs. HIV (**A**) penetrates the BBB by infected monocytes, and in most cases, the infected monocytes travel via the astrocytes, leading to neuroinflammation. AgNPs (**B**) have the potential to pass through the BBB due to their unique properties, such as their tunable size and shape. However, most antiretroviral drugs (**C**) have meager CNS-penetrating power. The conjugated AgNPs + ARDs navigate the BBB and interact with nervous tissues (**D**).

As illustrated in Figure 1, HIV has the ability to cross the blood–brain barrier (A), but a combined therapy of antiretroviral drugs (C) has been reported with low CNS-penetrating power. Similarly, all nanoparticles, including the silver nanoparticle (B), have higher penetrating power to the biological barriers. Hence, we hypothesized that combining silver nanoparticles (AgNP) with antiretroviral drugs (ARDs) would solve the issue of CNS penetration. We also hypothesized that silver nanoparticle-conjugated TDF can decrease the blood glucose level in STZ-induced hyperglycemic rats and improve cognitive functions due to the antidiabetic and antioxidant properties of silver nanoparticles. Therefore, the current study evaluated the consequences of the interaction of silver nanoparticles conjugated with tenofovir disoproxil fumarate on the hippocampal microanatomy and neuro-biomarkers in diabetic rats.

## 2. Results

### 2.1. The Results of AgNPs-TDF Characterisation

The AgNPs-TDF conjugate was investigated using ultraviolet–visible spectroscopy (UV–vis Spec), a transmission electron microscope of a high-resolution type (HR-TEM), and an energy-dispersive X-ray spectroscopic machine (EDX spec), respectively.

### 2.2. Ultraviolet–Visible Spectroscopy Analysis

**Figure 2:**
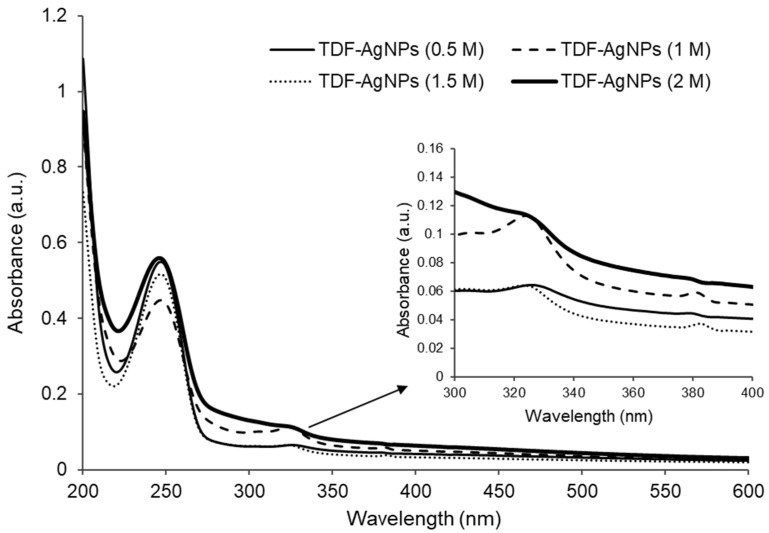
The ultraviolet-visible spectrum of the AgNPs-TDF at various concentrations. Adapted from a previous publication by Olojede et al. [23].

Figure 2: The UV–vis absorbance peaked at the range of 325–328 nm for the nanoconjugates (AgNPs-TDF) for the four concentrations (0.5 M, 1 M, 1.5 M, and 2 M) in this study, as shown in Figure 2. Additionally, Figure 2 shows that the conjugated AgNPs-TDF displayed absorbance peaks in two areas, found at 250 nm and between 325 and 328 nm. The observed absorbance peak values between 325 and 328 nm for the AgNPs-TDF are attributed to the localized surface plasmon resonance (SPR).

### 2.3. Transmission Electron Microscopy (TEM) Analysis

**Figure 3:**
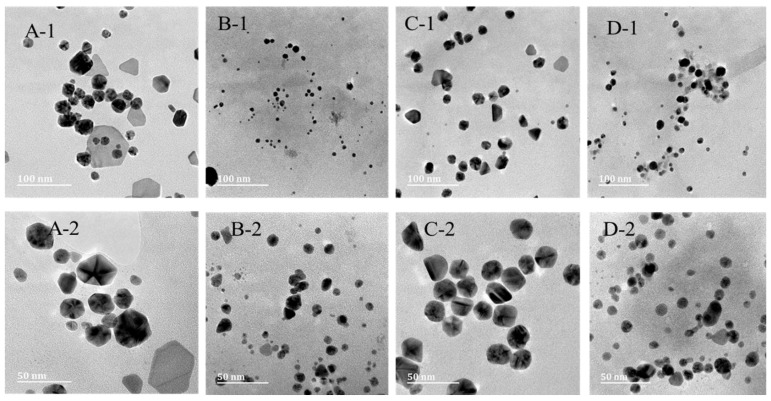
HR-TEM images of AgNPs-TDF (0.5 M) (**A**), AgNPs-TDF (1 M) (**B**), AgNPs-TDF (1.5 M) (**C**), and AgNPs-TDF (2 M) (**D**). (**A-1**) denotes 100 nm, (**A-2**) denotes 50 nm (0.5 M), (**B-1**) represents 100 nm, (**B-2**) represents 50 nm (1.0 M), (**C-1**) depicts 100 nm, (**C-2**) depicts 50 nm (1.5 M), (**D-1**) illustrates 100 nm, (**D-2**) illustrates 50 nm (2.0 M). Adapted from a previous publication by Olojede et al. [23].

Figure 3: The size and shape of the AgNPs-TDF were analyzed using TEM, as shown in Figure 1. The size of the nanoconjugated drug for a 0.5 M concentration ranged from 12 to 22 nm, and a concentration of 0.5 M exhibited a size range of 3–4 nm, with an average of 7 nm, while the 1 M concentration had an average particle size of 20 nm, although their sizes were within 13–32 nm. In addition, the concentrations of 1.5 M and 2 M showed sizes ranging from 13 to 32 nm (7 nm on average) and 5 to 23 nm (12 nm on average), respectively. The AgNPs-TDF of 1 M and 2 M concentration showed spherical shapes, while 0.5 M and 1.5 M displayed a mixture of spherical and hexagonal shapes.

### 2.4. Energy Dispersive X-Ray (EDX) Analysis

**Figure 4:**
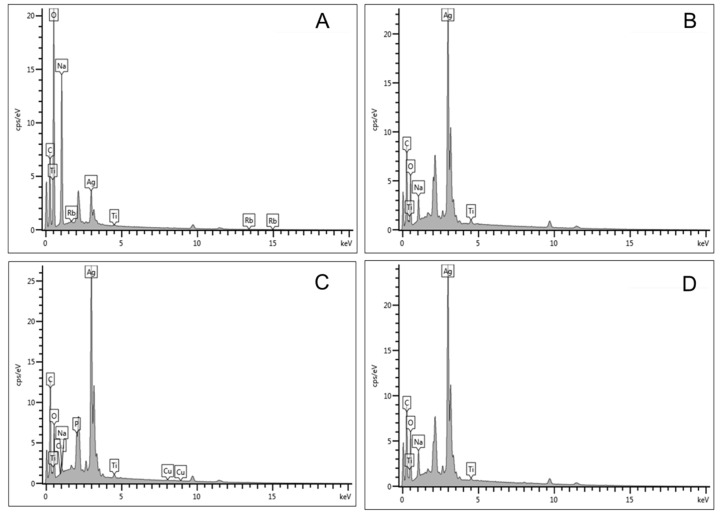
EDX spectra of AgNPs-TDF (0.5 M) (**A**), AgNPs-TDF (1 M) (**B**), AgNPs-TDF (1.5 M) (**C**), and AgNPs-TDF (2 M) (**D**). Adapted from a previous publication by Olojede et al. [23].

Figure 4: The elemental constituents of the formulated nanodrug were revealed by the energy-dispersive X-ray analysis, as shown in Figure 2. The results show that the AgNPs peaked at 3 keV. In addition, it was observed that the AgNPs were constituted with different percentages per weight. The weight of Ag in the AgNPs-TDF increased from 5.45% for 0.5 M to 38.59%, which is 1.5 M to 42.70% for 2 M and 43.03% for 1 M. The presence of elements including phosphorus, oxygen, and carbon signifies the proper conjugation of AgNPs and TDF.

Table 1: EDX analysis showing the elemental components of various concentrations of the AgNPs-TDF conjugate.

### 2.5. Effects of AgNPs-TDF on Bodyweight Difference and Blood Glucose Levels

**Figure 5:**
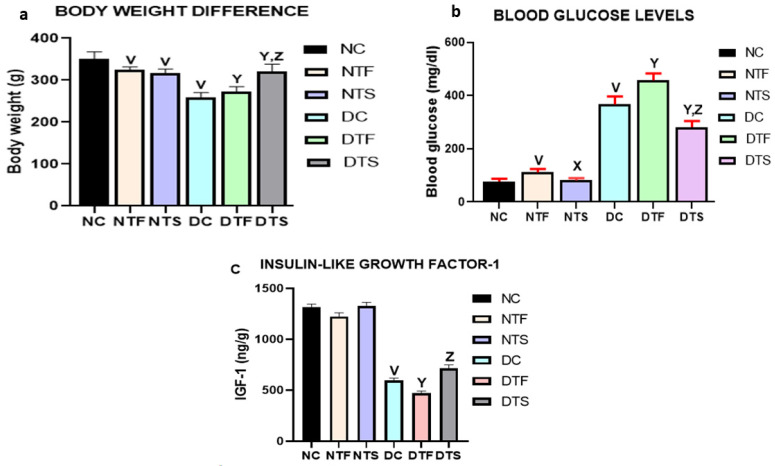
(**a**–**c**) Effects of AgNPs-TDF on bodyweight difference, blood glucose levels, and insulin-like growth factor-1 (IGF-1): ^V^ *p* < 0.05 vs. NC, ^X^ *p* < 0.05 vs. NTF, ^Y^ *p* < 0.05 vs. DC, ^Z^ *p* < 0.05 vs. DTF. NC = nondiabetic control, NTF = non-diabetic + TDF, NTS = non-diabetic + silver nanoparticles + TDF, DC = diabetic control, DTF = diabetic + TDF, DTS = diabetic + silver nanoparticles + TDF. Data are presented as mean ± SEM and are significant at *p* < 0.05. (n = 7).

Figure 5a,b: There was a significant reduction in body weight of all the treated animals in the non-diabetic and diabetic groups when compared with non-diabetic control (NC). The diabetic control group (DC) had a significant reduction in body weight compared to the normal control and other treated groups. Interestingly, the group treated with AgNPs-TDF (DTS) showed a significant improvement in body weight compared with all the diabetic-treated groups (DC and DTF) (Figure 5a). The weekly weight difference (dynamics of the animals) was measured and recorded during the experiment but there was no significant difference. Thus, the initial and final weight difference is discussed in this manuscript.

Similarly, the blood glucose level was significantly higher in the diabetic control (DC) compared with the other diabetic-treated groups (DTF and DTS). However, DTS had significantly reduced blood glucose levels compared with DC and DTF. Notably, there was a statistically significant decrease in the blood glucose level of NTS compared with the non-diabetic treated NTF (Figure 5b).

There was no significant difference in the concentration of IGF-1 in all the non-diabetic groups (NC, NTF, and NTS). The IGF-1 was significantly reduced in the diabetic control group (DC) compared with the normal control group (NC). There was a significant increase in the concentration of IGF-1 in group DTS compared with group DTF (Figure 5c).

### 2.6. Effects of AgNPs-TDF on Spatial Working Memory

**Figure 6:**
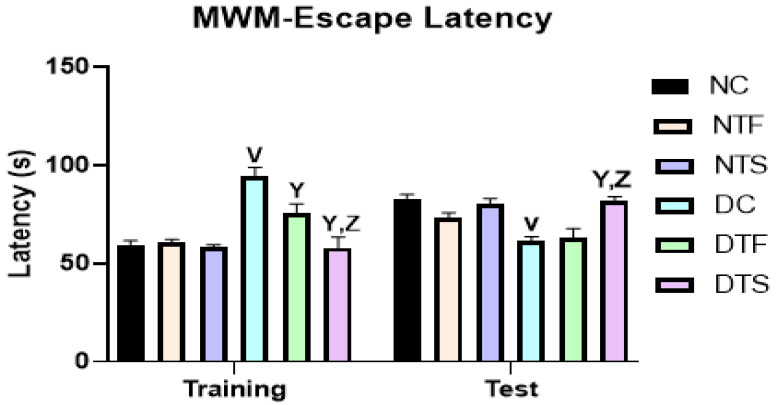
Effects of AgNPs-TDF on spatial working memory: ^V^ *p* < 0.001 vs. NC, ^Y^ *p* < 0.001 vs. DC, ^Z^ *p* < 0.001 vs. DTF. NC = nondiabetic control, NTF = non-diabetic + TDF, NTS = non-diabetic + silver nanoparticles + TDF, DC = diabetic control, DTF = diabetic + TDF, DTS = diabetic + silver nanoparticles + TDF. Data are presented as mean ± SEM and are significant at *p* < 0.001. (n = 7).

Figure 6: The cognitive functions of the experimental animals were assessed using Morris water maze escape latency. In training and testing, all the diabetic groups performed poorly compared with the non-diabetic groups. The test showed no difference between the TDF-treated and AgNPs-TDF-treated groups. However, the AgNPs-TDF-treated group spent more time in the quadrant previously containing the platform than the TDF-treated group and spent a significant amount of time in the quadrant compared with the diabetic control.

### 2.7. Effects of AgNPs-TDF on Locomotion and Explorative Behavior

**Figure 7:**
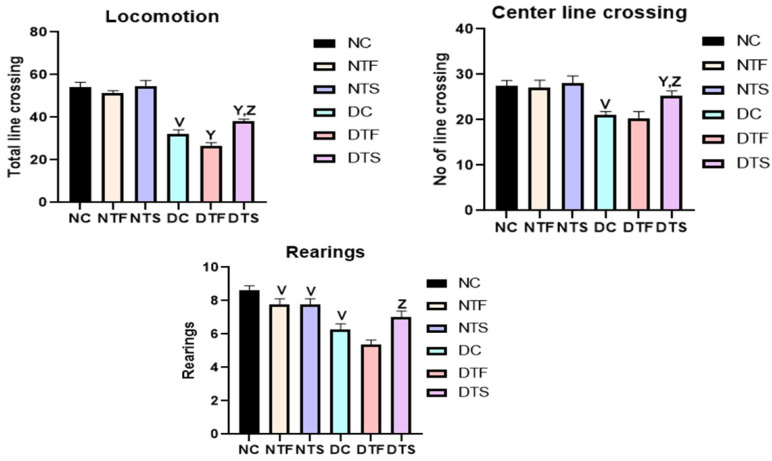
Effects of AgNPs-TDF on locomotion and explorative behavior: ^V^ *p* < 0.05 vs. NC, ^Y^ *p* < 0.05 vs. DC, ^Z^ *p* < 0.05 vs. DTF. NC = nondiabetic control, NTF = non-diabetic + TDF, NTS = non-diabetic + silver nanoparticles + TDF, DC = diabetic control, DTF = diabetic + TDF, DTS = diabetic + silver nanoparticles + TDF. Data are presented as mean ± SEM and are significant at *p* < 0.05.

Figure 7: The locomotion and explorative behavior of the experimental animals were assessed using an open-field test. The center line crossing, total line crossing, and rearing were used to examine the explorative and anxiety-like behavior. All the diabetic groups had a reduction in locomotion and explorative behaviors compared to the non-diabetic groups. Interestingly, the group treated with AgNPs-TDF significantly improved locomotion and exploration compared to the diabetic control and TDF-treated group.

### 2.8. Effects of AgNPs-TDF on Hippocampal Oxidative Stress Markers

Table 2: This table shows the results for the antioxidant parameters in the experimental animals. MDA was significantly higher in the diabetic rats and diabetic TDF-treated rats than in the other groups. The AgNPs-TDF-treated rats showed a significant decrease in MDA compared to the diabetic and diabetic TDF-treated rats. Similarly, the antioxidant parameters (SOD and GSH) were significantly increased in the diabetic AgNPs-TDF-treated rats compared to diabetic TDF-treated only.

### 2.9. Effects of AgNPs-TDF on Hippocampal Inflammatory Markers

Table 3: Neuroinflammation was assessed using TNF-α and IL-1β. The non-diabetic rats treated with TDF showed a significant increase in TNF-α compared to the control group. Similarly, the diabetic TDF-treated rats had a high-level TNF-α concentration compared to the diabetic control and AgNPs-TDF-treated rats. Interestingly, there was a significant decrease in the IL-1β and TNF-α concentrations in the AgNPs-TDF-treated rats compared to the diabetic control and TDF-treated only.

### 2.10. Effects of AgNPs-TDF on Hippocampal Ultrastructure—Neuronal Nuclear Membrane

**Figure 8:**
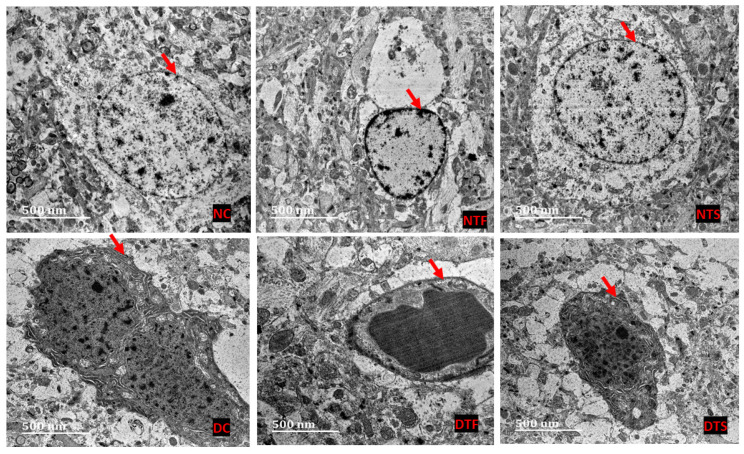
Effects of AgNPs-TDF on hippocampal ultrastructure neuronal nuclear membrane. NC = nondiabetic control, NTF = non-diabetic + TDF, NTS = non-diabetic + silver nanoparticles + TDF, DC = diabetic control, DTF = diabetic + TDF, DTS = diabetic + silver nanoparticles + TDF, red arrow = nuclear membrane. (n = 2).

Figure 8: The ultrastructure of the hippocampus was processed using TEM, and the results were analyzed. The nuclear membranes of the non-diabetic groups (NC, NTF, and NTS) showed a distinct oval-shaped nuclear membrane (NM) covering the nucleolus. The nuclear membrane (NM) was affected in all the diabetic groups (DC, DTF and DTS), showing a distorted and irregular-shaped membrane. However, the DTF group showed a similar oval-shaped nuclear membrane with vacuolating neoplasms.

### 2.11. Effects of AgNPs-TDF on Hippocampal Neuronal Nissl Bodies

**Figure 9:**
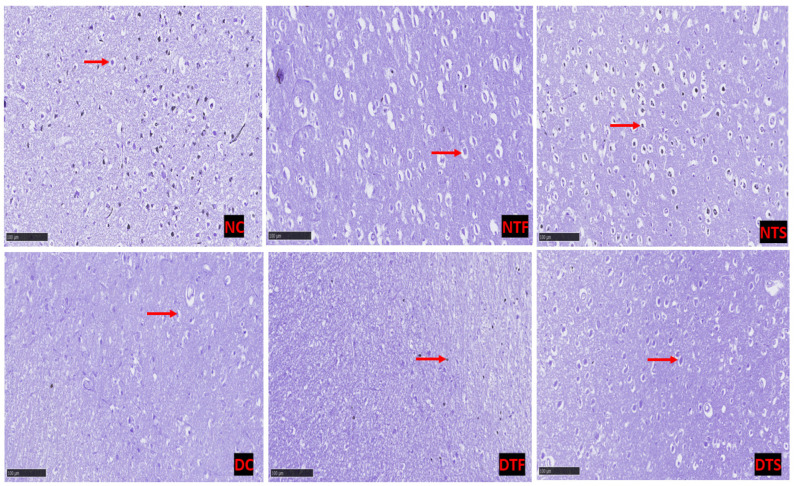
Effects of AgNPs-TDF on hippocampal neuronal Nissl bodies: NC = nondiabetic control, NTF = non-diabetic + TDF, NTS = non-diabetic + silver nanoparticles + TDF, DC = diabetic control, DTF = diabetic + TDF, DTS = diabetic + silver nanoparticles + TDF, red arrow = neurons. (n = 2).

Figure 9: Cresyl violet was used to stain the neuronal cell. The neurons of all the diabetic-treated rats showed chromophobic and pyknotic neuronal nuclei compared with the non-diabetic rats. Interestingly, the AgNPs-TDF showed a monomorphic pattern of dark-stained neuronal nuclei similar to the control group.

### 2.12. Effects of AgNPs-TDF on Hippocampal GFAP-Astrocytic Cells

**Figure 10:**
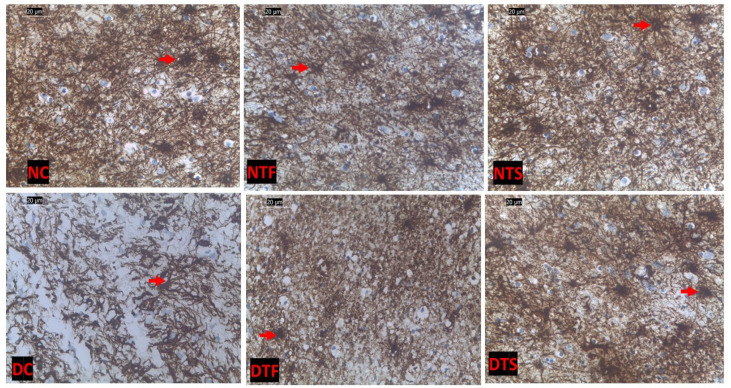
Effects of AgNPs-TDF on hippocampal GFAP-astrocytic cells: NC = nondiabetic control, NTF = non-diabetic + TDF, NTS = non-diabetic + silver nanoparticles + TDF, DC = diabetic control, DTF = diabetic + TDF, DTS = diabetic + silver nanoparticles + TDF, red arrow = astrocytic cell. (n = 2).

Figure 10: This shows the hippocampal-astrocytic cells in all the experimental rats. The normal control (NC) showed distinct astrocytes interconnected with the neighboring cells compared to the diabetic control (DC). There was a loss of distinct astrocytic cells in the diabetic rats treated with TDF compared to the rats treated with AgNPs-TDF.

### 2.13. Effects of AgNPs-TDF on Hippocampal Astrogliosis

**Figure 11:**
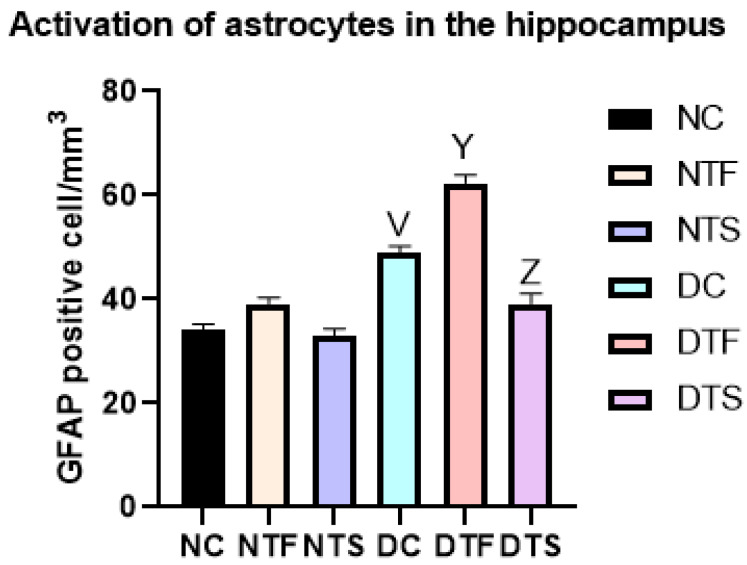
Effects of AgNPs-TDF on hippocampal GFAP-astrocytic cells: NC = nondiabetic control, NTF = non-diabetic + TDF, NTS = non-diabetic + silver nanoparticles + TDF, DC = diabetic control, DTF = diabetic + TDF, DTS = diabetic + silver nanoparticles + TDF. ^V^ *p* < 0.05 vs. NC, ^Y^ *p* < 0.05 vs. DC, ^Z^ *p* < 0.05 vs. DTF (n = 2).

Figure 11 shows the hippocampal astrogliosis in all the experimental rats. There was a significant increase in GFAP-positive astrocytic cells in all the diabetic-treated groups (DC, DTF, and DTS). Notably, the diabetic rats treated with TDF (DTF) significantly increased the number of activated astrocytes compared with the diabetic control (DC). However, in group DTS, the AgNPs-TDF significantly reduced the activation of GFAP-positive cells compared to the other diabetic groups.

## 3. Discussion

The nanoparticle drug delivery system has been viewed as an emerging field of nanomedicine that addresses issues related to biological penetration, challenges of targeted delivery, and therapeutic-associated toxicities. These functions have been ascribed to the properties of nanoparticles, which include tunable shape and size, as well as targeted and controlled release [23]. However, ample amounts of these nanoparticles have been loaded with several therapeutic agents and evaluated in vitro and in vivo with promising results. However, there is a dearth of information on the utilization of silver nanoparticles with proven anti-inflammatory, antidiabetic, antiviral, and antioxidant activities for their potential management of TDF-induced neurotoxicity. Hence, this study was conceived to unravel the mechanisms underlying the neuroprotective effects of silver nanoparticle-conjugated tenofovir disoproxil fumarate on the hippocampal microanatomy and neuro-biomarkers in diabetic rats.

Characterization represents an essential process to examine the peak value, size, shape, and elemental constituents of nanoparticles and conjugates. The observed absorbance peak values within two regions (between 325 and 328 nm and at 250 nm) for the AgNPs-TDF may be attributed to the localized surface plasmon resonance (SPR). Previously, Menon et al. [24] revealed that variations in the absorption peak and color changes are mostly determined by the SPR around the region of the electromagnetic spectrum. This indicates that the wavelength in the regions reported in this study was absorbed. Similarly, previous research has described the effect of the sensitivity of SPR to the shape and size changes of nanoparticles [25]. More so, both spherical and hexagonal nanoparticles were obtained in this investigation, which may be another factor for the absorption peak value noticed. Therefore, the SPR region was observed at 325 to 328 nm, possibly because of a combination of hexagonal and spherical-shaped nanoparticles, which agrees with a previous investigation [26]. In addition, the absorption peak of 250 nm recorded in this study has been previously documented in a similar spectrophotometric study that shows an absorption peak value of 259 nm for TDF, which signifies the proper conjugation of TDF to AgNPs [27].

The observed nanoparticle size ranging from 3 nm to 32 nm is similar to Van Dong and colleagues’ findings [28], who documented spherical-shaped AgNPs of sizes ranging between 4 and 40 nm. However, a mixture of hexagonal, rod, and spherical-shaped particles was observed in this study. However, the results acquired with 1 M and 2 M of AgNPs-TDF were predominantly spherical-shaped particles, which concurs with an investigation by Yang and co-workers [29], who documented spherical-shaped AgNPs of sizes within 30 nm. With an increased interest in AgNPs, experts have described particle size, especially within 30 nm, and shape, especially spherical-shaped, as important factors to consider when using AgNPs for pharmaceutical and biological uses [30].

The appropriate peak of AgNPs was noticed in this study, which aligns with the investigation performed by Mukherji and colleagues [31], who documented a peak of 3 keV for the AgNPs. In addition, the presence of the elemental constituents present in the TDF and AgNPs, such as phosphorus, carbon, and oxygen, signifies the proper conjugation of TDF with AgNPs. The remaining elements present in the results of the EDX were obtained from a reducing agent (trisodium citrate), while the copper grid employed to mount the samples gives copper, titanium, and rubidium, as found in the sample.

This study observed an elevated blood glucose level in diabetic rats treated with tenofovir disoproxil fumarate, confirming its metabolic disturbances reported in the literature [11,32]. Insulin resistance is common among HIV-positive people under antiretroviral treatment due to certain factors, including weight gain [32,33]. However, the moderate weight loss observed in this study suggests that other factors may play a role in metabolic disturbance rather than body weight gain, which is in line with other studies [33,34]. The silver nanoparticles possess antidiabetic effects, as reported by Alkaladi et al. [19]; in this study, the animals treated with the silver nanoparticles–TDF conjugate showed a consistent decrease in blood glucose level with improved body weight gain compared to the diabetic TDF-treated group.

This suggests that the silver nanoparticles improved glucose uptake and muscle cell sensitivity to blood glucose, thereby reducing blood glucose levels, similar to previous studies [35]. This effect was attributed to the special characteristics of silver nanoparticles, like large ratio of surface area to volume and antidiabetic properties, which act to inhibit carbohydrate metabolic enzymes (alpha-amylase and alpha-glucosidase) to reduce blood glucose levels [19,36].

Insulin-like growth factor systems and signaling pathways are involved in the differentiation, growth, and maintenance of neurons and supporting cells of the brain, as well as the prevention of neuronal apoptosis. However, damage to these systems is involved in neurodegenerative disorders like dementia and Alzheimer’s and metabolic dysfunctions [37,38]. The significant increase in IGF-1 levels in diabetes treatment suggests the capability of AgNP to enhance insulin secretion. This finding agrees with previous investigations that revealed the activities of AgNPs to lower blood sugar levels and enhance the secretion and sensitivity of insulin, which increase the level of IGF-1 in diabetic rats [13,39]. These findings altogether suggest that an increase in the IGF-1 level is responsible for the decrease in blood glucose level observed in this experiment.

The prevalence of neurocognitive disorders is still high in HIV-positive people despite the numerous benefits of combined therapy, including TDF [3]. The current study showed an extent of neurocognitive dysfunction in the tenofovir-treated group. However, the animals treated with the AgNPs-TDF conjugate showed improved neurocognitive function through restoring various alterations in the hippocampal ultrastructure and improving memory assessment in diabetic rats. This observation could be attributed to the ability of silver nanoparticles to deliver a low-dose and effective drug release, reducing TDF’s neurotoxic effect [40,41].

Further investigation in this current study revealed the metabolic disturbance of TDF via oxidative stress markers. Glutathione, which is involved in tissue repair and combating stress-induced tissue damage, was depleted in the diabetic TDF-treated animals with increased lipid peroxidation. This result correlates with a previous study [42]. However, the silver nanoparticles ameliorated the oxidative effects by blocking TDF’s excessive reactive oxygen species (ROS) production via increased antioxidant glutathione [43].

In the present study, the neurotoxic effect was evidenced by the high concentration of neuroinflammatory markers (IL-1β and TNF-α) in the hippocampus of the TDF-treated animals. Several studies have reported the neuroinflammatory effects of TDF [44,45]. However, the conjugated silver nanoparticles with tenofovir drastically reduced the concentration of neuroinflammatory markers. Studies have reported the anti-inflammatory properties of silver nanoparticles with green synthesis [38,46], and the mechanism of these anti-inflammatory activities has been linked with the ability of silver nanoparticles to inhibit some selected cytokine agents, such as IL-6 and IL-1β [47]. More evidence has emerged that AgNPs lower the initiation of transcription pathways involving TNFR1/NF-KB, directly reducing TNF-α‘s inflammatory response [48].

The observed distorted neuronal nuclear membrane and astrocytic cells in the hippocampus of diabetic rats, as seen in the results of this study, could suggest a deleterious effect of diabetes mellitus on hippocampus cytoarchitecture. Empirical evidence has indicated that the hippocampus is one of the most essential parts of the brain susceptible to metabolic dysfunction like diabetes mellitus [49]. A similar in vivo study on an animal model revealed diabetic-induced neuronal nuclear membrane alterations, reduction in cell body diameter, neuronal nuclear reduction, and decreased neuronal density in the hippocampus [50]. In addition, the loss of connection between the surrounding neuroglia and neurons in diabetic rats indicates hippocampal injury, which is consistent with a previous study that documented neuroglia degeneration and detachment in an experimental diabetic rat model [51,52]. Consequently, it leads to neuronal death because the function of the neuroglia is to support and insulate neurons; once the connection is lost, the neurons are susceptible to death [52]. This diabetic-induced hippocampal damage and neuronal cell death were attributed to insulin resistance, chronic hyperglycemia, and deficits in hippocampal insulin signaling, which leads to cognitive and behavioral [53]

Furthermore, alteration to the neuronal cell membrane in diabetic rats treated with tenofovir disoproxil fumarate indicates damage to the hippocampus.

An investigation by Fields, Swinton [3] documented alterations to the hippocampal structures in an in vivo mice model, similar to the result of this present study, although not in a diabetic condition. Several studies have attributed mitochondrial dysfunction and accumulation of reactive oxygen species as mechanisms responsible for tenofovir-induced hippocampal neuronal alteration and, consequently, neurocognitive impairments [54,55,56]. Additionally, the chromophobic and pyknotic neuronal cells’ hippocampal neurons noticed with tenofovir administration in this study concur with a similar investigation that reported negative Nissl-stained hippocampal neuronal cells following administration of an antiretroviral combination containing tenofovir in diabetic rats [57]. In addition, the loss of GFAP-astrocytic cells in the TDF-treated rats observed indicates astrogliosis, as reported by previous studies [58,59]. These reports, together, suggest a hippocampal neuron distortion with the administration of tenofovir in diabetic or non-diabetic conditions.

Interestingly, a monomorphic pattern of dark-stained neuronal nuclei and restoration in the loss of astrocytic cells following administration of AgNPs-TDF in this study indicates some neuroprotective impact of nanoparticles synthesized from silver. Although many studies have suggested that silver nanoparticles are neurotoxic in vitro and in vivo [60,61,62], this is on account that smaller particle sizes like silver nanoparticles can easily navigate the blood–brain barrier to gain access to and interact with brain tissue [63]. However, emerging evidence has revealed that the neurotoxicity of silver nanoparticles largely depends on several factors, such as the concentration of silver nitrate, surface coating, reducing agent, particle size, particle shape, level of exposure, agglomeration, and method of preparation [64]. The neuroprotective impact obtained may be attributed to the smaller size and spherical shape of silver nanoparticles used in this study, which elicits its antioxidant, anti-diabetic, and anti-inflammatory properties to restore some hippocampal alterations. This study agrees with Lawal et al. [13] findings, who reported improved neurocognitive deficits through silver nanoparticles’ antioxidant, antidiabetic, and anti-inflammatory properties loaded with antiretroviral drugs. This finding suggests that the biological activities of silver nanoparticles could be effectively exploited in the delivery of antiretroviral drugs by taking care of factors associated with its toxicity profile to enhance the efficacy of these agents in managing neurocognitive deficits.

## 4. Materials and Methods

### 4.1. Material

The TDF (300 mg) was purchased at the Dis-Chem pharmacy in Durban, South Africa. Streptozotocin (STZ), trisodium citrate, sodium hydroxide, and silver nitrate (AgNO_3_) were purchased from Sigma-Aldrich Company, Johannesburg, South Africa. Enzyme-linked immunosorbent assay (ELISA) kits, TNF-α (cat no: E-EL-R0019), interleukin (IL)-1β (cat no: E-EL-R0012), and primary antibody (Anti-GFAP: AB10062) and secondary antibody (Goat Anti-mouse IGg: AB150113) (Elab Science Biotechnology Co., Ltd., Houston, TX, USA) were sourced from BIOCOM Africa (pty), Ltd., Centurion, South Africa.

### 4.2. Animal

Forty-two (42) adult male Sprague-Dawley rats weighing between (250 ± 13 g) were acquired from the Biomedical Research Unit of the University of KwaZulu-Natal (UKZN-BRU). All the animals were kept in the animal laboratory in highly ventilated plastic cages 24 cm in height, 36 cm wide, and 52 cm in length. They were allowed to feed (standard rat pellets) and drink freely. The room temperature was maintained at 24–26 °C with a 12:12 light:dark cycle.

### 4.3. Ethical Approval

The animal laboratory procedures and study protocol were approved by the Research Ethical Committee of the University of KwaZulu-Natal with AREC number (AREC/044/19D). All the procedures were carried out at the Biomedical Research Unit (BRU) and overseen by a certified veterinarian (SAVC registration number SAVC D14/11254).

The animals received humane care based on the Care and Use of Laboratory Animals guide provided by the National Institute of Health (NIH Publications No. 80-23).

### 4.4. Experimental Design

The rats were left to acclimatize for seven (7) days; after that, they were divided randomly into diabetic and non-diabetic groups. Both the non-diabetic and diabetic groups were further divided into three (3) subgroups (n = 7). The non-diabetic groups 1–3 (designated as non-diabetic control (NC)) received 0.5 mL/100 g distilled water per os (p.o.), non-diabetic + tenofovir (NTF) were administered 26.8 mg/kg/bw TDF p.o., and non-diabetic + tenofovir + silver nanoparticles (NTS) were administered 6.7 mg/kg AgNPs-TDF intraperitoneally (i.p.), respectively.

Similarly, the diabetic groups 4–6 (designated as diabetic control (DC)) were administered 0.5 mL/100 g distilled water per os (p.o.), diabetic + tenofovir (DTF) received 26.8 mg/kg/bw TDF p.o., and diabetic + tenofovir + silver nanoparticles (DTS) received 6.7 mg/kg AgNPs-TDF intraperitoneally (i.p.), respectively.

The given dose was determined by our previous studies [42,65], and the animal dose for TDF was estimated from the human equivalent dose (HED), as described by the United States Food and Drug Administration (FDA) [66]. The administration of the testing agents began after diabetes induction. Oral administration was given daily for eight weeks, and intraperitoneal injection was completed 5/7 days for 8 weeks. The TDF was administered orally, while the AgNPs-TDF was administered intraperitoneally. The reason for administering AgNPs-TDF intraperitoneally was informed by the need for several physiological barriers and hurdles, such as gastric mucosal composition, gastric motility, and gastric pH, before the loaded drug can reach the target cell.

Moreover, under the 3 Rs and 5 Freedom of Animal Research Ethics, we reduced the IP administration from 7 days a week to 5 days. Behavioral assessments were conducted on the second resting day of the animals to cater to the animals’ welfare.

### 4.5. Induction of Type II Diabetes Mellitus in Experimental Rats

After seven (7) days of acclimatization, each rat’s baseline body weight and blood glucose were measured and recorded. After that, hyperglycemia with insulin resistance was induced using the fructose–streptozotocin (STZ) rat model described by Wilson and Islam [67]. Briefly, the diabetic groups (4–6) received a fructose solution (10% fructose solution *ad libitum*) for two weeks. On the final day of administering fructose, the rats were fasted overnight, and freshly prepared 40 mg/Kg/bw STZ dissolved in 0.9% NaCl with 100 mM sodium citrate buffer (pH 4.5) was given via the intraperitoneal route. In contrast, the control rats were given the same volume of citrate buffer (vehicle). Fasting blood glucose levels ≥ 200 mg/dL were an indication of diabetes in the rats.

#### 4.5.1. Synthesis of AgNPs and Formulations of AgNPs-TDF

Silver nitrate (AgNO_3_) and trisodium citrate (TSC) were employed to synthesize the silver nanoparticles. An aqueous solution of 0.3 M AgNO_3_ and four concentrations (0.5 M, 1 M, 1.5 M, and 2.0 M) of TSC were prepared. The silver nanoparticles were prepared by mixing the two solutions (20 mL of AgNO_3_ and 20 mL of concentrations of TSC in a ratio of 1:1) in a beaker while continuing to stir for 5 min at 90 °C. The pH of 10.5 was adjusted using NaOH. The resultant solution was stirred continuously until the color changed from colorless to amber color. A solution of 0.35 M of tenofovir dyspraxia fumarate (TDF) was prepared by dissolving the crystal form of TDF in 1 M of NaOH. The already synthesized AgNPs were then mixed with a solution of TDF (50 mL of TDF and 100 mL of AgNPs in a ratio of 1:2) and sonicated at a frequency of 20–40 kHz for 15 min. Lastly, the AgNPs-TDF supernatant was analyzed with UV–vis spec, and the percentage incorporated was determined and calculated. The full protocol has been previously described [23]. The efficiency of the percentage of AgNPs-TDF incorporated was calculated as follows:

$$\%\;IE=\frac{Q_{2}-Q_{1}}{Q_{1}}\times 100\;=85.00\;\pm \;0.0\%.$$

where *Q*_1_ = quantity of unincorporated drug and *Q*_2_ = total amount of drug coupled with the silver nanoparticles.

#### 4.5.2. Characterization of AgNPs and AgNPs-TDF

The characterization of the AgNPs and AgNPs-TDF was reported previously by [23]. Briefly, the formulation of the AgNPs-TDF conjugate was confirmed with ultraviolet–visible (UV–vis) spectroscopy (Shimadzu MultSpec-1501, Shimadzu Corporation, Tokyo, Japan) at an absorption peak from 325 to 328 nm. Fourier transform infrared (FTIR) spectroscopy (Perkin-Elmer Universal ATR spectrometer, Waltham, MA, USA) was used to identify the various functional groups in the AgNPs-TDF conjugates. The presence of C-N and O-H functional groups in the AgNPs-TDF confirmed that AgNPs-TDF was conjugated. The size and morphology of the AgNPs-TDF were examined by a high-resolution transmission electron microscope (HR-TEM, JEOL 2100, Chiba, Japan) operated at a voltage of 200 kV. The HR-TEM revealed the nanoparticle shape and size of the AgNPs-TDF conjugates to be spherical, and the mean particle sizes were 12 nm to 22 nm.

A field emission scanning electron microscope (FESEM, Carl Zeiss, Oberkochen, Germany), operated at a voltage of 5 kV with energy dispersive X-ray (EDX, Aztec Analysis Software, version 6.0 Southampton, UK), was used to determine the elemental components, and this confirmed the presence of silver in the AgNPs-TDF.

### 4.6. Blood Glucose and Body Weight Difference Measurement

After diabetic induction and during the drug administration, fasting blood glucose was obtained using a glucometer (Sigma-Aldrich, Durban, South Africa) via the tail vein of the rats on a weekly basis. The body weight difference was measured using a sensitive weighing balance.

### 4.7. Behavioral Studies

#### 4.7.1. Morris Water Maze for Spatial Working Memory

On the 7th week of administration, the spatial working memory was evaluated using the Morris water maze, as described by the previous studies of Greish et al. and Morris [68,69]. Briefly, the instrument is made up of a standard circular swimming pool 100 cm in diameter half-filled with water of about 22–25 °C. Each of the rats was assigned 120 s to locate the platform that was hidden 2 cm below the water level, and the animal that could not find the escape platform after 120 s of the trial time was calmly led to the platform and allowed to be on the platform for a few seconds. In addition, the latency was recorded.

After two days, the test was repeated on each animal, following the same procedure as the training trial. The percentage of time spent swimming in the quadrant previously containing the platform was recorded and calculated to determine the spatial working memory.

The animals that spent longer in the quadrant previously containing the platform were considered to have learned and retained memory.

#### 4.7.2. Open Field Test for Locomotion and Explorative Behavior

The rats were assessed for locomotion and explorative behavior through the open-field test during the resting day of administration in the 7th week. The open-field apparatus was set up according to previous studies [70,71]. Briefly, using a measuring diameter 70 cm long × 70 cm wide × 35 cm high with several 15 cm × 15 cm squares, a large rectangular measuring box was used. The rats were positioned in the center of the square and examined for 5 min. Parameters like total line cross, center line cross, and rearing were obtained. The exploration of the animals was assessed using the number of times the animal stood up on its posterior limbs (rearing). This behavior imitates an activity of space exploration of a new environment [72].

### 4.8. Euthanasia Procedure and Tissue Harvesting

Twenty-four (24) hours after the last administration, the experimental animals were euthanized using isoflurane in a closed container. The animals were observed until signs of breathing stopped, and the animals (n = 2) were humanely euthanized according to standard protocol by the AREC-approved guidelines. Tissue samples were collected for histological and immunohistochemical analysis. Two (2) out of seven (7) animals were used for histological and immunohistochemical analysis. This was done to prevent other brain tissues used for biochemical analysis from being contaminated with fixatives.

### 4.9. Determination of Antioxidant Levels and Other Biochemical Analyses

The hippocampal tissue was homogenized with 0.1 M phosphate buffer (pH 7.5) using a polytron homogenizer. The homogenate was centrifuged at 15,000 rpm for 5 min in a Centrikon H-401 (Kontron, Munich, Germany) centrifuge at 4 °C. After centrifugation, the upper part was collected and analyzed. The following antioxidant superoxide dismutase (SOD) and catalase (CAT) were analyzed according to previous protocols [65,73] reduced glutathione (GSH) was determined using Ellman’s protocol [66] and the malondialdehyde (MDA) level was determined using the method of Mkhwanazi et al. [67].

### 4.10. Determination of Inflammatory Biomarkers

The tumor necrosis factor-α (TNF-α) and interleukin (IL)-1β concentrations were quantified in the hippocampal homogenates using their specific ELISA kits (Elab Science Biotechnology Co., Ltd., Houston, TX, USA) based on the instructions and protocols of the manufacturer.

### 4.11. Histopathological and Immunohistological Studies

The brain was carefully removed and weighed, then post-fixed in 4% paraformaldehyde for 48 h. The hippocampus was dissected from the brain using a rat atlas and processed for histological analysis. The tissue was infiltrated with 30% sucrose for 3 days at 4 °C. The paraffin-embedded tissue was sectioned at 5 μm using a Leica RM 2255 microtome and stained according to Bancroft and Stevens protocol [74] for hematoxylin and eosin (H&E) and Nissl staining.

The remaining sections of the hippocampus used immunohistochemical analysis. Firstly, the sections were washed in PBL and pre-incubated in 5% goat serum (0.1 M with 0.4% Triton X-100, 1 M PBL and albumen) for 1 h at 4 °C, then directly incubated in the primary antibody (anti-GFAP) diluted in PBSA-Triton at a ratio of 1:5000. After that, the sections were washed in PBST (2 × 10 min) and incubated in a secondary antibody for 2 h at room temperature. They were then rinsed in PBST (2 × 10 min) and incubated with an avidin–biotin complex for 2 h, followed by several washes (1 × 10 min in PBST and 2 × 10 min in Tris buffer (0.05 M, PH 7.6)). Peroxidase activity detection was carried out with 3-3′ diaminobenzidine. The immunoreactive reaction was stopped by washing the sections once in 0.1 M Tris buffer (10 min) and twice in 0.1 M PBS (10 min). The sections were dehydrated in progressive ethanol baths, cleared in 2 successive xylene baths, mounted onto gelatin-coated slides, and coverslipped. The primary antibody (Anti-GFAP: AB10062) and secondary antibody (Goat Anti-mouse IGg: AB150113) (Elab Science Biotechnology Co., Ltd., Houston, TX, USA) were sourced from BIOCOM Africa (pty), Ltd.

### 4.12. Transmission Electron Microscopic Analysis of Hippocampal Tissue

The hippocampal tissue sectioned at 1 mm^3^ was post-fixed in glutaraldehyde (12 h) and washed (3 × 10 min) in phosphate buffer. After that, it was transferred to osmium tetroxide (2 h) and washed again in phosphate buffer (3 × 5 min). The sections were dehydrated in varying degrees of acetone-containing solutions (30%, 50%, 75%, and 100% for 5 min) and embedded in Durcopan (Fluka). The hippocampal tissue was re-sectioned (1 μm in thickness) using an ultramicrotome (Leica Ultracut R), contracted by lead and uranyl acetate, and finally examined with a transmission electron microscope.

### 4.13. Statistical Analysis

All the data were analyzed using Graph Pad Prism 8 for Windows (GraphPad Software San Diego, CA, USA). *p* < 0.05 was considered statistically significant and presented as mean ± SEM. The differences between the means were compared using one-way analysis (ANOVA), followed by Tukey’s multiple comparison test to determine the statistical significance between the groups.

G Power Software (Version: 3.1.9.7.) was used to calculate the number of animals based on the difference in the means between two independent groups to arrive at a total sample size of 42 animals. This was based on the literature, and the minimum number of animals per group for behavioral studies was eight (8) to have statistically significant values.

## 5. Conclusions

This study provides invaluable insights into the adverse effects of diabetes mellitus and tenofovir disoproxil fumarate on hippocampal microanatomy and neuro-biomarker alteration. It underscores the neuroprotective role of silver nanoparticle-capped tenofovir disoproxil fumarate, which restores various alterations on the hippocampal microstructures and cognitive functions in rats via silver nanoparticles’ antioxidant and anti-inflammatory activities. In addition, the unique properties of silver nanoparticles could be effectively exploited in the delivery of antiretroviral drugs to the CNS to enhance the efficacy of these agents in managing neurocognitive deficits.

## Figures and Tables

**Table 1:** Energy dispersive X-ray analysis shows elemental components of the various concentrations of AgNPs-TDF conjugate. C = carbon, O = oxygen, Na = sodium, Ti = titanium, Ag = silver, Rb = rubidium, Cu = copper, and P = phosphorus.

Elements	Sample 0.5 M (Wt%)	Sample 1.0 M (Wt%)	Sample 1.5 M (Wt%)	Sample 2.0 M (Wt%)
C	30.64	25.32	30.76	25.51
O	49.76	27.63	27.51	27.76
Na	13.95	3.40	0.47	3.69
Ti	0.09	0.62	0.52	0.34
Ag	5.45	43.03	38.59	42.70
Rb	0.11	00.00	00.00	0.00
Cu	00.00	00.00	0.30	0.00
P	00.00	00.00	1.85	0.00
Total	100	100	100	100

**Table 2:** Effects of AgNPs-TDF on hippocampal oxidative stress markers: ^V^ *p* < 0.05 vs. NC, ^X^ *p* < 0.05 vs. NTF, ^Y^ *p* < 0.05 vs. DC, ^Z^ *p* < 0.05 vs. DTF. NC = nondiabetic control, NTF = non-diabetic + TDF, NTS = non-diabetic + silver nanoparticles + TDF, DC = diabetic control, DTF = diabetic + TDF, DTS = diabetic + silver nanoparticles + TDF. Data are presented as mean ± SEM and are significant at *p* < 0.05. (n = 5).

Hippocampal Oxidative Markers	Animal Grouping					
	NC	NTF	NTS	DC	DTF	DTS
MDA (nmol/mg)	30 ± 1.34	41.42 ± 1.57 ^V^	30.46 ± 1.71 ^X^	65.73 ± 1.79 ^V^	72.64 ± 1.47 ^Y^	45.30 ± 0.97 ^Y,Z^
SOD (u/mg)	10.06 ± 0.23	9.26 ± 0.22	9.19 ± 0.39	4.87 ± 0.17 ^V^	3.54 ± 0.32 ^Y^	6.29 ± 0.18 ^Y,Z^
CAT (nmol/mg)	16.50 ± 0.70	12.83 ± 0.78	14.12 ± 0.56 ^X^	7.51 ± 0.36 ^V^	7.70 ± 0.59	9.93 ± 0.42 ^Y^
GSH (nmol/mg)	91.26 ± 3.12	76.38 ± 3.92	86.23 ± 3.30 ^V^	49.02 ± 1.77 ^V^	37.34 ± 1.03 ^Y^	62.85 ± 2.16 ^Y,Z^

**Table 3:** Effects of AgNPs-TDF on hippocampal inflammatory markers: ^V^ *p* < 0.05 vs. NC, ^Y^ *p* < 0.05 vs. DC, ^Z^ *p* < 0.05 vs. DTF. NC = nondiabetic control, NTF = non-diabetic + TDF, NTS = non-diabetic + silver nanoparticles + TDF, DC = diabetic control, DTF = diabetic + TDF, DTS = diabetic + silver nanoparticles + TDF. Data are presented as mean ± SEM and are significant at *p* < 0.05. (n = 5).

Groups/Parameters	TNF-α (pg/mL)	IL-1β (pg/mL) (n = 5)
NC	185.4 ± 4.71	35.19 ± 1.00
NTF	234.50 ± 6.29 ^V^	43.48 ± 2.83
NTS	204.60 ± 6.09	40.55 ± 2.52
DC	339.90 ± 13.85 ^V^	72.84 ± 1.98 ^V^
DTF	412.30 ± 14.14 ^Y^	88.96 ± 1.76 ^Y^
DTS	309.50 ± 4.908 ^Y,Z^	55.13 ± 1.53 ^Y,Z^

## Data Availability

The raw data supporting the conclusions of this article will be made available by the authors upon request.
